# hSAGEing: An Improved SAGE-Based Software for Identification of Human Tissue-Specific or Common Tumor Markers and Suppressors

**DOI:** 10.1371/journal.pone.0014369

**Published:** 2010-12-17

**Authors:** Cheng-Hong Yang, Li-Yeh Chuang, Tsung-Mu Shih, Hsueh-Wei Chang

**Affiliations:** 1 Department of Electronic Engineering, National Kaohsiung University of Applied Sciences, Kaohsiung, Taiwan; 2 Department of Network Systems, Toko University, Chiayi, Taiwan; 3 Department of Chemical Engineering, I-Shou University, Kaohsiung, Taiwan; 4 Department of Biomedical Science and Environmental Biology, Kaohsiung Medical University, Kaohsiung, Taiwan; 5 College of Pharmacy, Graduate Institute of Natural Products, Kaohsiung Medical University, Kaohsiung, Taiwan; 6 Center of Excellence for Environmental Medicine, Kaohsiung Medical University, Kaohsiung, Taiwan; 7 Cancer Center, Kaohsiung Medical University Hospital, Kaohsiung Medical University, Kaohsiung, Taiwan; King's College London, United Kingdom

## Abstract

**Background:**

SAGE (serial analysis of gene expression) is a powerful method of analyzing gene expression for the entire transcriptome. There are currently many well-developed SAGE tools. However, the cross-comparison of different tissues is seldom addressed, thus limiting the identification of common- and tissue-specific tumor markers.

**Methodology/Principal Findings:**

To improve the SAGE mining methods, we propose a novel function for cross-tissue comparison of SAGE data by combining the mathematical set theory and logic with a unique “multi-pool method” that analyzes multiple pools of pair-wise case controls individually. When all the settings are in “inclusion”, the common SAGE tag sequences are mined. When one tissue type is in “inclusion” and the other types of tissues are not in “inclusion”, the selected tissue-specific SAGE tag sequences are generated. They are displayed in tags-per-million (TPM) and fold values, as well as visually displayed in four kinds of scales in a color gradient pattern. In the fold visualization display, the top scores of the SAGE tag sequences are provided, along with cluster plots. A user-defined matrix file is designed for cross-tissue comparison by selecting libraries from publically available databases or user-defined libraries.

**Conclusions/Significance:**

The hSAGEing tool provides a combination of friendly cross-tissue analysis and an interface for comparing SAGE libraries for the first time. Some up- or down-regulated genes with tissue-specific or common tumor markers and suppressors are identified computationally. The tool is useful and convenient for *in silico* cancer transcriptomic studies and is freely available at http://bio.kuas.edu.tw/hSAGEing

## Introduction

Serial analysis of gene expression (SAGE) [1] can quantitatively evaluate expression profiles of the entire transcriptome without prior sequence information [2], [3], [4], [5] in contrast to the microarrays. SAGE provides high sensitivity for mRNAs of low abundance [6], [7] and detects slight differences in expression levels between samples, providing information necessary for the identification of new tumor biomarkers and suppressors [8], [9], [10], [11], [12].

SAGE usually generates a huge amount of experimental data, i.e., SAGE tag sequences and their counts (including noisy and redundant data). It is necessary to extract and arrange the relevant information in SAGE data to find a key SAGE tag (or a set of SAGE tags). Many publicly available bioinformatics tools [13], [14], [15], [16], [17], [18], [19], [20] were developed to address this point (mentioned in detail in the discussion section later). However, they fail to provide the cross-tissue comparison of gene expressions, which means that the mined SAGE tag sequences representing the tumor marker candidates in some tissues can not simultaneously be cross-compared to the tumor marker candidates in other tissues. Moreover, matrix data is usually not provided in SAGE. Without matrix data, the screening history of SAGE library components is not recorded for repeated checking if needed. Users are unable to recall members of the original SAGE libraries that were previously screened and analyzed, and thus reproducibility of the analysis is reduced. Accordingly, simultaneous mining and matrix data generating for tissue specific- and common-tumor marker candidates among several tumor and control tissue types is still challenging.

In light of these caveats, we propose a new function that analyzes SAGE data by combining the mathematical set theory and logic [21] with a unique “multi-pool method” designed to analyze multiple pools of pair-wise case control comparisons individually. Set theory [21] is the mathematics method that studies sets, which are collections of objects. Theoretically, any type of object can be collected into a set for set theory application. With the help of set theory, the common and the tissue-specific SAGE tag sequences can be mined by this multi-pool method.

This work presents a novel greenware, hSAGEing that provides a friendly gene expression mining interface for analysis, comparison, and visualization of the built-in human SAGE data. We developed custom matrix creation, cross-tissue comparison, and analysis functions, and a visualization platform for the SAGE libraries, SAGE tags-to-genes, and SAGE tag-to-libraries. Gene expression differences between many SAGE library pools can be identified, and the tool provides common and tissue-specific SAGE tag sequences for tumor markers.

## Results

In this study, we propose an integrated platform for analyzing SAGE data. The main graphical user interface (GUI) provides the four functions describe below.

### Matrix data creator

**Figure 1:**
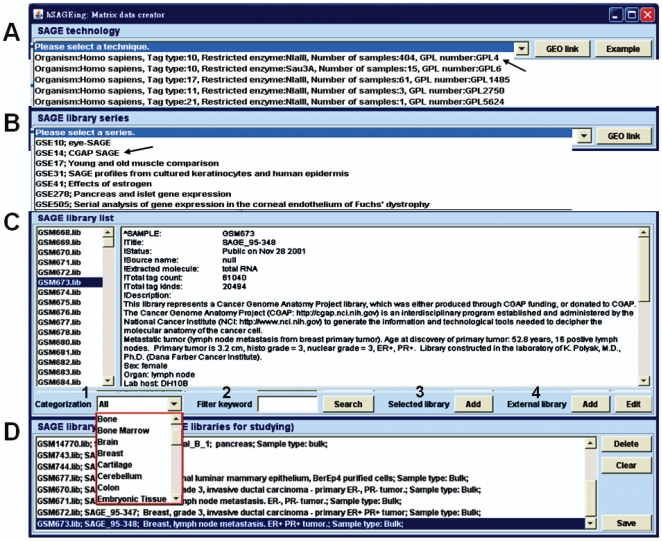
Screenshot of the matrix data creator function. This interface contains four functions: (A) SAGE technology (GPL-series), (B) SAGE library series (GSE-series), (C) SAGE library list (GSM-series), and (D) SAGE library pool (in which SAGE libraries can be added). The three-layer categorization of all libraries is shown in (A), (B), and (C). (C) Four functions are provided: 1) categorization, 2) keyword filtering, 3) library selection, and 4) external libraries. (D) The compilation of a custom matrix data file is shown. This matrix file can be edited and saved for further analysis in the cross-extraction function.

Three-layer categorization from top to bottom for SAGE data such as SAGE technology, SAGE library series, and SAGE library described later (system database in Section of Methods) are provided in three separate windows (Figures 1A, 1B, and 1C, respectively). Figure 1A shows that five SAGE technologies are available for download from GEO of NCBI. Once the technique of interest is selected, i.e., “organism: *Homo sapiens*, Tag type: 10, Restricted enzyme: NlaIII, Number of samples: 404, GPL number: GPL4” (as indicated by the arrow line), hSAGEing is able to provide the detailed information by clicking the external “GEO link” (http://www.ncbi.nlm.nih.gov/geo/query/acc.cgi?acc=GPL4). Subsequently, the corresponding SAGE library series, i.e., GSE10, GSE14, GSE17, GSE31, GSE41, GSE278, GSE505, and others (Figure 1B), are presented. If different SAGE technologies are chosen, the external GEO link and its corresponding SAGE library series are different (not shown).

Once the SAGE library series is selected, i.e., GSE14; CGAP SAGE (indicated by the arrow line in Figure 1B), the information for the selected SAGE library series is available by clicking the (http://www.ncbi.nlm.nih.gov/geo/query/acc.cgi?acc=GSE14) external “GEO link”. The library list contained in this SAGE library series (GSE14) is accessible by clicking on the left side of Figure 1C.

**Figure 2 pone-0014369-g002:**
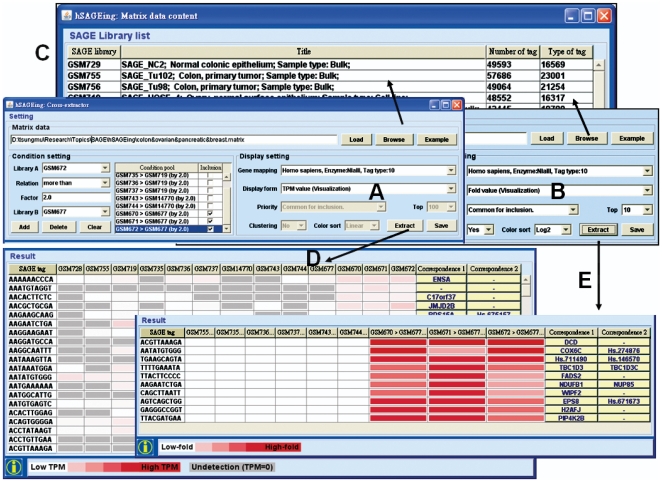
Screenshot of the cross-extraction function. The main interface includes: (A) and (B) settings, (C) matrix data content for SAGE library list, and (D) and (E) visualization results. (A) and (B) The custom matrix file loader provides three functions, the matrix data upload, condition settings, and display settings. In the condition pool of the condition setting, common tags are chosen for all the conditions with “inclusion  =  Yes”. When the conditions are set to “inclusion  =  No”, the filtered SAGE tags are not included in the common tags. Therefore, both tissue-specific and common tumor markers can be mined (see details in the Result section). (D) Result contains the filtered SAGE tags, the gene expression level for each SAGE tag in each SAGE library, and the mapped gene symbols listed under correspondence 1 and 2 (right side of Figures 2D and 2E). The expression level (TPM) is displayed as a color gradient, i.e., white means low level, red means high level, and gray means undetectable in the SAGE library. (E) The result contains the filtered SAGE tags, the gene expression fold (ratio of TPM of two libraries) within one pair of SAGE libraries (in the condition pool) for each SAGE tag. It also contains the mapped gene symbols.

Once the SAGE library of interest is selected, such as GSM673.lib (indicated by the black background on the left side of Figure 1C), the detailed information for this SAGE library is provided (the right side of Figure 1C) via mining of the local database retrieved from GEO of NCBI (GSM673 is renamed GSM383828 by GEO; http://www.ncbi.nlm.nih.gov/geo/query/acc.cgi?acc=GSM383828). The SAGE library list is categorized into many tissue types as described in SAGE Genie [14] to provide a selection of tissue-specific SAGE libraries for selected SAGE library series, such as adipose, adrenal cortex, adrenal medulla, bone, bone marrow, brain, breast, cartilage, cerebellum, cervix, colon, ear (inner), embryonic tissue, endocrine, esophagus, eye, gastrointestinal tract, germ cell, head and neck, heart, kidney, limb, liver, lung, lymph node, lymphoreticular, mammary gland, muscle, nerves, ovary, pancreas…and so on (shown in the pull-down window of Figure 1C-1). A filter keyword function (Figure 1C-2) provides a text mining for the SAGE library selection from the information shown in the right main window of Figure 1C. The hSAGEing tool provides a single (clicking one item on the SAGE library) or multiple selections (clicking with shift or control keys pressed down) for SAGE libraries. A user simply clicks on the box (Figure 1C-[Fig pone-0014369-g002] 3) to put the selected libraries into the SAGE library pool (Figure 1D).

The hSAGEing system provides 979 SAGE libraries for selection in the setting “SAGE technology: Organism  =  *Homo sapiens*, tag type  = 10, restricted enzyme  =  NlaIII; SAGE library series: CGAP SAGE [14]  =  GSE14:CGAP SAGE series” (data not shown). Clicking on “brain” in the categorization window, the number of related SAGE libraries is reduced down to 254. Afterward, the “medulloblastoma” inputs for retrieval of related SAGE libraries are reduced to 59. Users can thus select SAGE libraries of interest more easily.

External SAGE libraries (Figure 1C-[Fig pone-0014369-g002] [Fig pone-0014369-g003] 4), i.e., user-defined and other SAGE data, are also accepted for SAGE analysis. Users can upload an external SAGE library by clicking on the “add” button in Figure 1C-[Fig pone-0014369-g002] [Fig pone-0014369-g003] 4 into the SAGE library pool. Clicking the “edit” button in Figure 1C-[Fig pone-0014369-g002] [Fig pone-0014369-g003] 4 shows the format and an example of how to set up a SAGE library file (.lib).

Before saving a file (Figure 1D), users can select other SAGE libraries again from SAGE library list (left side of Figure 1C) or from external SAGE files (Figure 1C-[Fig pone-0014369-g002] [Fig pone-0014369-g003] 4). All the selected SAGE libraries are put into a pool for editing (deleting and clearing) and this file is then saved in a certain file format for further analysis by the matrix data creator module.

When users select the “example” box on the right side of Figure 1A, a step-by-step demonstration of how to produce the matrix data file is shown (Figures 1A to 1D, also shown on-line or in the user manual).

### Cross-tissue extraction

Clicking on the “example” button in Figures 2A and 2B brings up a detailed tutorial. Clicking on the “load” button, the matrix data file name (such as the “colon&ovarian&pancreatic&breast.matrix” provided by the hSAGEing tool as the built-in example file) and SAGE libraries (listed in the pull-down window for libraries A and B) appear in the “matrix data” and “condition setting” fields, respectively (Figures 2A and 2B). Detailed information for each SAGE library, i.e., SAGE library name, title, number of tag, and type of tag (Figure 2C) is provided by clicking on the “browse” button. The SAGE libraries listed in the example matrix file can be selected in the pull-down windows for the “libraries A and B” in the condition setting (left side of Figure 2A) and compared to each other with adjustable “factor” (fold value) and “relation” settings (i.e., more than, less than, or not in). Finally, the selected libraries and their relationships are shown in the box “condition pool”.

All information for these SAGE libraries from the example file, “colon&ovarian&pancreatic&breast.matrix” as described above, is shown in the “condition pool” of Figure 2 and in Table S3B. Details of the SAGE libraries listed in Figure 2C are provided in Table S3A.

#### Example of breast-specific tumor markers

**Figure 3 pone-0014369-g003:**
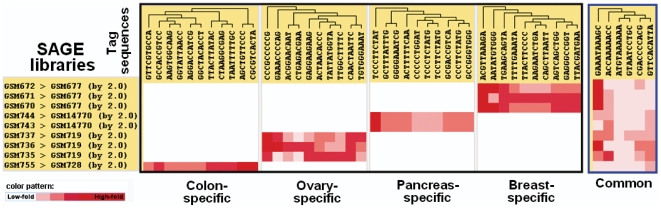
Demonstration of SAGE mining for tissue-specific and common tumor markers. This plot integrates five individual figures, i.e., five different conditions generate these five figures (colon-, ovary-, pancreas-, breast-specific and common tumor markers) one-by-one. The SAGE libraries in the GSM series are listed in Table S3 and in the conditioning pool of Figure 2A, although the “inclusion” in the condition setting is different when different tissue-specific tumor markers are mined.

When the “inclusion” buttons are marked by clicking, the mining of candidate tags is based on the selected conditions (e.g., breast cancer and normal SAGE libraries: GSM670>GSM677, GSM671>GSM677, and GSM672>GSM677, as shown in “condition pool” in Figure 2A) and does not include the mining of candidate tags from conditions that are presently not selected (e.g., colon, ovary, and pancreas of normal and cancer SAGE libraries: GSM755>GSM728; GSM735>GSM719, GSM736>GSM719, GSM737>GSM719; and GSM743>GSM14770, GSM744>GSM14770, respectively). Accordingly, the SAGE mining provides the SAGE tags for breast cancer-specific tumor candidates without homolog to the SAGE tags for other types of cancers. Clicking on the “extract” button (right side of Figures 2A or 2B), the SAGE tags (such as ACGTTAAAGA, AATATGTGGG, TGAAGCAGTA…and TTACGATGAA; from left to right in Figure 3) representing breast cancer-specific tumor candidates are shown.

#### Example of pancreas-specific tumor markers

Similarly, pancreas-specific tumor markers in Figure 3 were mined with the setting “inclusion  =  Yes” for filter conditions 5 and 6 (Table S3B) and at “inclusion  =  No” for any other filter conditions (Table S3B), i.e., (inclusion  =  Yes) GSM743>GSM14770, and GSM744>GSM14770 *vs.* (inclusion  =  No) GSM670>GSM677, GSM671>GSM677, GSM672>GSM677, GSM755>GSM728, GSM735>GSM719, GSM736>GSM719, GSM737>GSM719.

#### Example of ovary- or colon-specific tumor markers

The ovary-specific tumor markers shown in Figure 3 were mined with the setting “inclusion  =  Yes” for filter conditions 2, 3, and 4 and “inclusion  =  No” for any other filter conditions shown in Table S3B, while colon-specific tumor markers were mined with “inclusion  =  Yes” for filter condition 1 and “inclusion  =  No” for the other filter conditions shown in Table S3B.

#### Example of common-tumor markers for breast, pancreas, ovary, and colon cancers

When all “inclusion” boxes are selected (Table S3B), the common tumor markers for colon, ovary, pancreas, and breast cancers are shown in Figure 3. The entire operational steps for all tissue-specific and common tumor markers are provided in the user manual. Although only the top 10 candidates are provided, this number can be adjusted up to the top 100.

#### Example of common-tumor suppressors for breast, pancreas, ovary, and colon cancers

Similarly, common tumor suppressor candidates can be mined when all cancer groups are set to “less than” their corresponding normal controls, i.e., a setting not shown in Table S3B. For example, the SAGE tag sequence GGCCCTGAGC, which is matched to the gene candidates for POLR2L and MEA1, is mined under the setting (GSM743<GSM4770, GSM744<GSM4770, GSM670<GSM677, GSM671<GSM677, GSM672<GSM677, GSM755<GSM728, GSM735<GSM719, GSM736<GSM719, and GSM737<GSM719 (inclusion  =  Yes)) (shown in user manual).

### Display setting

In the display setting, hSAGEing provides four output types for cross-extraction functions, namely TPM value, TPM value (visualization), fold value, and fold value (visualization). TPM and fold values provide the (digital) number for tag counts and tag ratio, respectively. The TPM value (visualization) and the fold value (visualization) provide a graphic depiction of TPM and fold values as shown in Figures 2A and 2B, respectively. The results shown in Figures 2D and 2E can be saved. The visualization uses gradient color patterns (color sorting) of different scales, e.g., linear, log2, log10, and square root.

### Output

In the output for the TPM value (visualization), the TPM for many tag sequences (in different rows) for each library (in different columns, e.g., Table S3A) is listed individually (Figure 2D). In the output for fold value (visualization), the fold value for many tag sequences (in different rows) for any paired libraries in each row of the condition pool in Figure 2A or Table S3B is listed individually in different columns in Figure 2E. On the left side of Figure 2E, the filter conditions 1 to 6 in Table S3B (colon-, ovary-, and pancreas-specific markers) are set to “inclusion  =  No” and the color patterns of the tags are almost white (low fold). The tags for the breast-specific tumor candidates have high fold values (shown in red color), and conditions 7 to 9 were set to “inclusion  =  Yes” in Table S3B or Figure 2A). Therefore, the SAGE tags with breast-specific tumor markers rather than the colon-, ovary-, and pancreas-specific tumor markers are mined successfully (right side of Figure 3). Similarly, the SAGE tags with colon-, ovary-, or pancreas-specific tumor markers can be mined individually (left side of Figure 3). The SAGE tags for their common tumor markers can also be extracted (right side of Figure 3). A clustering function for the “fold value (visualization)” is provided optionally in the display setting (Figure 2B). The clustering results can be saved in a file, an example of which is shown in Figure 3.

After choosing the SAGE technology (as described in Figure 1A) from the gene mapping of the display setting (Figures 2A and 2B), hSAGEing provides an external link to identify the possible gene candidates belonging to each tag, e.g. the corresponding 1 (GenBank of NCBI) and corresponding 2 (Unigene of NCBI) located on the right side of Figures 2D and 2E. When the gene mapping is not selected, these external links are not provided.

### Tag-to-gene

**Figure 4:**
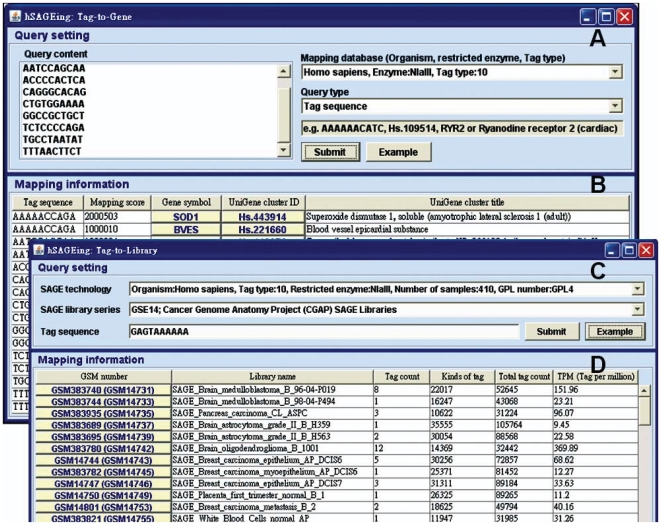
Screenshot of the tag-to-gene and tag-to-library functions. The interface is divided into (A and C) query settings and (B and D) mapping information. (A) The input contains the query content, the mapping database, and the query types. (B) The output contains a list of mapping information (tag sequence, mapping score, gene symbol, UniGene cluster ID, and UniGene cluster title). (C) The input contains the SAGE technology, the SAGE library series, and the tag sequence. (D) The output contains a list of mapping information (GSM number, library name, tag count, kinds of tag, total tag count and TPM. Since the GEO recently renames some SAGE libraries, we expressed the SAGE library name as a new GSM no. (old GSM no.).

Tag-to-gene functions provided by hSAGEing are the tag sequence input and the corresponding output of the mapping score, gene symbol, UniGene cluster ID, and UniGene cluster title based on the local built-in tag-to-gene database. In Figures 4A and 4B, nine tag sequences, i.e., AAAAACCAGA (not shown, in the top of the pull-down window for query content), AATCCAGCAA, ACCCCACTCA, CAGGGCACAG, CTGTGGAAAA, GGCCGCTGCT, TCTCCCCAGA, TGCCTAATAT, and TTTAACTTCT, are used as examples. A mapping database (right side of Figure 4A) is selected, in our example Organism: *Homo sapiens*, Restricted enzyme: NlaIII, and Tag type: 10, and a mapping information is provided (Figure 4B). In the shown example, the tag sequence AAAAACCAGA hits the SOD1 and BVES genes (the top two rows in Figure 4B). External links to GenBank and Unigene cluster ID are provided via a mouse-click. Moreover, the query type in the pull-down window provides the gene symbol, UniGene cluster ID, and UniGene cluster title search in addition to tag sequences.

### Tag-to-library

In Figure 4C, two search levels, namely the SAGE technology and SAGE library series, are provided in the query setting for tag-to-library when the tag sequence is input. The output for the tag-to-library function provides mapping information for the SAGE library list. This output includes the GSM number, library name, tag count, kinds of tag (with unrepeated tag sequences), total tag count, and TPM. In the example of the tag sequence GAGTAAAAAA input (Figure 4C), the software hit 117 SAGE libraries (only 12 libraries are partly shown in Figure 4D) from a total of 214 CGAP SAGE libraries when the query was set to “GPL4 and GSE14” (Figure 4C) for SAGE technology and SAGE library series, respectively.

## Discussion

Excluding TAGmapper [16] and DicoverySpace [17], which are no longer maintained, feature comparisons are made as shown in Table 1 between hSAGEing and other available SAGE software tools. Several improvements in hSAGEing are listed as follows:

**Table 1:** Comprehensive assessment of hSAGEing and related software tools.

Software tools Functions	hSAGEing(this study)	SAGEmap *1 [13]	SAGE Genie [14]	Mouse SAGE Site [15]	ACTG [18]	SQUAT [19]	SAGE in GEO [20]	Extract SAGE [22]
Database (organisms)	Human(GEO/updated CGAP)	Some	Human , mouse	Mouse	Human, mouse	Avian, marine, human	Some	Human (old CGAPmap)
External library input (SAGE libraries nos./ can be saved in database)	Yes (many/saved )	–*2	Yes (only 2/not saved )	–	Yes (only for tag sequences)	Yes	Yes (many/saved )	Yes (many/saved )
Library information browser	Yes	Yes	Yes	Yes	–	–	Yes	Yes
Library anatomical (tissue) viewer	Yes	–	Yes	Yes	–	–	–	–
Library finder by keyword	Yes	–	Yes	–	–	Yes	–	–
File saving- text	Yes (.txt)	Yes (.htm)	Yes (.htm)	Yes (.htm)	Yes (email)	Yes (.txt)	Yes (.txt)	Yes (.mgf)
File saving- image	Yes (.png)	–	–	–	–	Yes	–	–
Matrix file creator	User-defined	–	–	–	–	User-defined	Built-in (fixed)	–
User defined library construction (format & tutorial provided; file save)	Yes (.lib similar to .xls)	–	–	–	–	–	Yes (web deposit)	Yes (.lib)
Filter genes by set theory conditions	Yes	–	–	–	–	–	–	Yes (but no “inclusion”) *6
Analyses of gene expression in multiple SAGE libraries compared to their controls	Yes (many pools) [each case *vs.* each control]	Yes (2 pools) *3	Yes (2 pools) *4,*5	Yes (2 pools) *3	–	–	–	Yes (many pools)
Cross-tissue comparison of gene expression	Yes (tissue-specific & common tags)	–	–	–	–	–	–	Yes (common tags only)
Visualization for gene expression level	Yes (4 scales)	–	Yes (1 scale)	–	–	–	– (only for array)	Yes (1 scale)
Clustering for SAGE tag sequences	Yes	–	–	–	–	–	– (only in array)	–
Tag-to-library	Yes	Yes	–	Yes	–	Yes	–	–
Tag-to-gene	Yes	Yes	Yes	Yes	Yes	Yes	–	Yes (no link)

*1. Undergoing restructuring.

*2. Symbol “–“ indicates no function.

*3. Average counts of all cases *vs.* all controls.

*4. Similar to *3 but the same tissue of case and control is compared (like SAV in SAGE Genie), different tissues are not compared to each other; *5. DGED in SAGE Genie: It identifies those genes that are expressed at different levels in two pools of human libraries, e.g., if three libraries (1, 2, and 3) are included and Pools A and B are cases and controls, respectively. Several conditions are performed as follows: 1 in pool A *vs.* 1, 2 or 3 in pool B; 2 in pool A *vs.* 1, 2 or 3 in pool B; 3 in pool A *vs.* 1, 2 or 3 in pool B. Demonstrations for *3, *4, and

*5 are provided in the user manual.

*6. See “Inclusion” at Figuection.

### Improved input and output in hSAGEing

Some SAGE mining tools, e.g., ACTG [18] (http://retina.med.harvard.edu/ACTG/) and SQUAT [19] (http://bsmc.insa-lyon.fr/squat/login.php), do not provide library browsing function. This function is however implemented in SAGEmap [13] (http://www.ncbi.nlm.nih.gov/projects/SAGE/), SAGE Genie [14] (http://cgap.nci.nih.gov/SAGE), mouse SAGE site [14] (http://mouse.img.cas.cz/sage/), GEO [20] (http://www.ncbi.nlm.nih.gov/geo/), Extract-SAGE [22] (ftp://sage@bio.kuas.edu.tw/Extract-SAGE.zip), and hSAGEing. Only hSAGEing and SAGE Genie provide functions for SAGE library selection using the tissue viewer (Figure 1C-1) and keyword input (Figure 1C-2). Among the tools in Table 1, only hSAGEing, GEO and Extract-SAGE allow input from external libraries that can be saved in the database. In addition to the original database, only hSAGEing, GEO, and Extract-SAGE provide a tutorial and format for user defined library construction.

The output results for all the tools listed in Table 1 can be saved in text or html format. However, only hSAGEing, SQUAT, and GEO provide for the saving of image files. Currently, hSAGEing provides a built-in SAGE database that relies solely on the human genome, but it is laid out for future expansion to other species.

### Improved matrix file creator in hSAGEing

Table 1 shows that only hSAGEing, SQUAT, and GEO provide a function for matrix file creation. GEO provides a matrix file in its built-in database, which cannot be adjusted to suit a specific user requirement. In contrast, hSAGEing and SQUAT provide a user-defined way for matrix file creation. SQUAT generates the matrix file by only inputting SAGE tag sequences and their corresponding gene expression levels. It is deficient in other SAGE information like the descriptions for sample and tissue type.

### Improved cross-tissue extraction in hSAGEing

The platforms of SAGEmap, SAGE Genie, and mouse SAGE site are restricted to the analysis of only two pools of library data. They compare two pools containing the average data of the cases and controls. SAGEmap and mouse SAGE are based on the average SAGE count of the pool A, which is then compared to the average SAGE count of the pool B even when different tissue types are chosen. Tissue-specific issues are ignored.

SAGE Genie uses two types of SAGE mining tools, i.e., the SAGE Anatomic Viewer (SAV, http://cgap-stage.nci.nih.gov/SAGE/AnatomicViewer) and the SAGE Digital Gene Expression Displayer (DGED, http://cgap-stage.nci.nih.gov/SAGE/SDGED_Wizard?METHOD=SS10,LS10&ORG=Hs). SAV (shown in *4 of Table 1) is based on the average SAGE counts of pool A compared to the average SAGE counts of pool B for each tissue, i.e., average counts for tissue A (libraries a1–a3, cases) in pool A are compared to tissue A (libraries a4–a6, controls) in pool B, for tissue C (libraries c1–c3, cases) in pool A are compared to tissue C (libraries c4–c6, controls) in pool B, and so on. Further examples are shown in the user manual built-in the tool. Hence, the tissue-specific SAGE tag sequences are provided in SAV based on the comparison of the average count in cases and controls for each tissue. However, the built-in SAGE libraries are fixed in each tissue, i.e., the library members in each tissue cannot be changed, and thus users-defined SAGE mining cannot be performed. Contrary to SAV, DGED (shown in *5 Table 1) is based on the individual SAGE tag count comparison between pool A and pool B for each library. However, cases and controls for different tissues are also compared (see the example in *5 of Table 1). Accordingly, the tissue-specific issue is not considered in DGED.

In hSAGEing, SAGE mining is based on the set theory and the user-defined matrix file. To our knowledge, this use of the set theory in SAGE related mining tools is novel. The filter condition for the set theory is setting on individual case SAGE library compared to individual control SAGE library. Many comparisons are acceptable by introducing pair-wise case control settings (as shown in Table S3B and the condition pool in Figure 2A). Thus, the SAGE tag sequences for each condition are individually mined. When the “inclusion” boxes for all conditions are marked (see Table S3B), the common SAGE tag sequences are provided (right side of Figure 3). Clicking on conditions belonging to the same tissue but un-clicking the conditions belonging to other tissues, the system provides a tissue-specific SAGE tag sequence for the selected tissue (Figure 3) (detailed operations are described in the Results section for Figures 2 and 3). In contrast, Extract-SAGE only provides the SAGE tag for common tumor markers without creation of a matrix file (Figure 1) and “inclusion” functions described in the condition pool (Figure 2A). Thus, hSAGEing provides a novel function for identifying common- and tissue-specific tumor markers based on SAGE tag sequences.

Visualization for SAGE mining tools is provided only by SAGE Genie and hSAGEing (Table 1). The visualization in hSAGEing is presented in a gradient color pattern (color sorting) of different scales, i.e., linear, log2, log10, and square root rather than the single scale chosen in SAGE Genie and Extract-SAGE. This design improves the case of selection of closed count values from amongst different pools. The clustering of the mined SAGE tag sequences also enhances the visualization in hSAGEing.

In the display form of the “Fold value”, some calculations for the relative fold values may face a special condition that zero may found in some SAGE tags, making the fold value becomes infinity. Therefore, we follow the criteria described in the Mouse SAGE (http://mouse.img.cas.cz/sage/help.cgi?subj=compare) [14], i.e., the fold factor is computed simply by dividing normalized tag count in pool #2 by normalized tag count in pool #1. Therefore, we adjust the zero value of the denominator into the lowest value among the SAGE tags in the SAGE library. While the numerator is zero, we still keep the as zero.

### Improved tag-to-gene and tag-to-library functions in hSAGEing

Except for GEO, all SAGE tools in Table 1 can execute tag-to-gene functions. Only hSAGEing, SAGEmap, Mouse SAGE, SQUAT, and Extract-SAGE, however, implement the tag-to-library function. SAGEmap, mouse SAGE, and SQUAT provide a tag-to-library function relying on the retrieval of all built-in SAGE libraries. In contrast, hSAGEing provides a tag-to-library function that is only limited by the user-defined or user-selected SAGE libraries, i.e., it is only based on the SAGE technology and SAGE library series (Figure 4C) that users have chosen. In other words, hSAGEing provides “pure” libraries without the “noise”.

### Comparison of the tumor marker candidates mining from hSAGEing to literature

For breast cancer, four tags such as ACGTTAAAGA, AATATGTGGG, AGTCAGCTGG, and TTACGATGAA listed in the Figure 4 are identified to the genes for dermcidin (DCD), cytochrome c oxidase subunit Vic (COX6C), epidermal growth factor receptor pathway substrate 8 (EPS8), and phosphatidylinositol-5-phosphate 4-kinase, type II, beta (PIP4K2B), respectively. These genes had been reported to be the breast cancer tumor markers in literature. For examples, DCD is overexpressed in some invasive breast carcinomas [23], COX6C is important in discriminating hormone responsive breast cancer [24], [25], EPS8 is identified as novel putative oncogenes in breast cancer [26], and overexpression of PIP4K2B is important in the development and/or progression of breast cancer [27]. For pancreatic cancer, the tag GGGGAAATCG, belonged to the thymosin beta-10 (TMSB10) gene, is reported to upregulate in human pancreatic carcinoma, but not in control pancreatic tissue [28]. For ovarian cancer, the tags CAACTAATTC and TGTGGGAAAT belong to the clusterin (CLU) and secretory leukocyte peptidase inhibitor (SLPI) genes, respectively. Overexpression of CLU was found in human ovarian carcinoma [29] and was correlated with impairedsurvival [30]. High levels of serum SLPI were significantly elevated in ovarian cancer patients compared with benign control [31]. For colon cancer, the tag AGGACCATCG, belonged to the keratin 18 (KRT18) gene, is reported to be overexpressed in SW613-S human colon carcinoma cell line, compared to nontumorigenic control [32]. Based on these examinations, the hSAGEing-predicted tumor markers are consistent with the literature.

### Future directions

Recently, high-throughput DNA sequencing methods has applied to the RNA-Seq [33] to revolutionize the analysis for both mapping and quantifying transcriptomes. It is based on deep sequencing of transcripts of interests to generate millions of short reads for analysis. These short reads are in units of“transcripts per million” and allows comparison between expression of different transcripts, which is similar to the tags in SAGE data. In future, our proposed hSAGEing tool may be further improved to help for the RNA-seq analysis.

### Conclusion

Taken together, this study demonstrates that hSAGEing is more efficient, informative and versatile than other SAGE mining tools, especially with regard to the SAGE library information browser, library tissue viewer, keyword-centric library finder, matrix file creator, user-defined library construction, set theory-based settings, cross-tissue comparison for multiple pools of SAGE libraries, clustering display, four-scale color gradient patterns, file output for text and image saving, and the tag-to-gene and tag-to-library functions. Therefore, hSAGEing is novel in its use of multiple simultaneous comparisons of SAGE libraries (such as cancer vs. normal tissue comparisons over multiple tissues) which increase its efficiency over existing software. It also has more visualization options for fold-change and absolute expression across samples and comparisons than current software.

## Methods

### Implementation and availability

**Figure 5:**
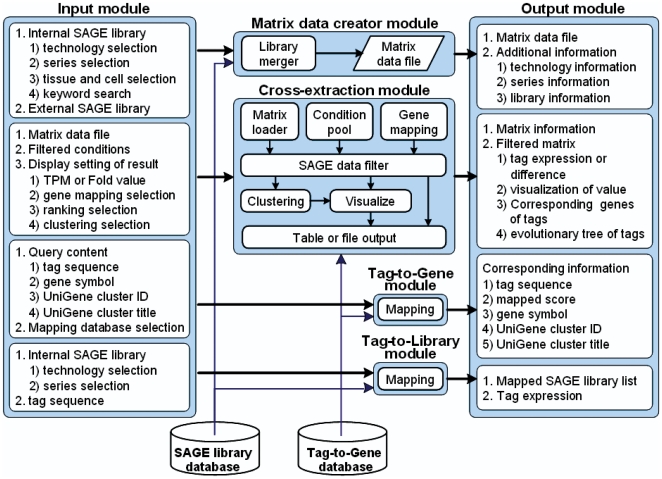
System structure and flowchart of hSAGEing.

The hSAGEing program is a Java-based tool application for comprehensive SAGE data analysis. A demonstration of the tool applet with free access to the tools described later and its user manual is available at http://bio.kuas.edu.tw/sage/hSAGEing.zip and http://bio.kuas.edu.tw/sage/hSAGEing-usermanual.pdf, respectively. The flow chart of this system (listed in Figure 5) including the modules for input, matrix data creator, cross-extraction, tag-to-gene, tag-to-library, and output are described in detail later.

### System database

The database contains the SAGE libraries and the tag-to-gene database. All SAGE libraries for *Homo sapiens* (about 979) were retrieved from Gene expression omnibus (GEO) [20] of NCBI (ftp://ftp.ncbi.nih.gov/pub/geo/), and include normal and cancer tissue/cell lines for many tissue types. The SAGE libraries consist of three categories, namely the SAGE technology, the SAGE library series, and the SAGE library from top to bottom (Figure S1); these levels are based on GEO criteria [20].

In the top-level category SAGE technology, all SAGE libraries are sorted by tag sequence lengths (10-, 11-, 17-, or 21-bp) and restriction enzymes (NlaIII or Sau3A), e.g., “Tag type: 10, Restriction enzyme: NlaIII”. In the second-level SAGE library series, some series were included, e.g., GEO: eye-SAGE (http://www.ncbi.nlm.nih.gov/geo/query/acc.cgi?acc=GSE10), the collections for human retinal and RPE SAGE libraries HRPE1, HPR1, HPR2, and HMAC2. The lowest-level SAGE library contains the sample information and gene expression data for various cell and tissue samples. Although only the *Homo sapiens* SAGE libraries are built-in, the system is designed to be expandable and accepts user defined SAGE data and NCBI SAGE libraries from other species for SAGE analysis (described later in the Result section).

The tag-to-gene database for the *Homo sapiens* SAGE libraries is downloaded from the SAGEmap [13] (ftp://ftp.ncbi.nlm.nih.gov/pub/sage/mappings/) (Table S1). The SAGE annotation information such as gene marker, matched score, UniGene cluster ID, and UniGene cluster title for the corresponding SAGE tag sequences are included.

### Input module

Both internal and external SAGE libraries are acceptable as input for the matrix data creator module. In the cross-extraction module, the matrix data creator module-generated matrix data file is processed based on some filter and optional selection settings. The tag abundance value is transformed into the TPM value (tags per million) for each SAGE tag in each SAGE library in order to ensure that the analysis is performed with an equal base line in these libraries. The tag-to-gene module accepts keywords and database selections (database title, tag type, restriction enzyme type, and latest update) as query inputs (Table S1). In the tag-to-library module, tag sequences are matched to the internal SAGE libraries.

### Matrix data creator module

Using the “library merger” function, all selected libraries in the SAGE library pool can be merged into a gene expression matrix S for output of a “matrix data file”. In this matrix S, each column represents different SAGE libraries and each row represents different SAGE tags. The element sij represents the gene expression (in TPM) of SAGE tag i in SAGE library j. This step proved useful for enhancing the search and estimating the gene expression profile.

### Cross-extraction module

Three input parameters, the matrix data loader, the condition pool, and the gene mapping, are fed into the SAGE data filter, the core of the data processing unit. The process performs the following steps: The matrix data generated by the matrix data creator module are uploaded via the matrix loader. The matrix data are screened to generate a filtered matrix based on the set theory [21] and logic criteria listed in the condition pool shown in formula (1).
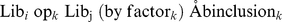
(1)


Lib*_i_* and Lib*_j_*: matrix files contained in the SAGE libraries.

op*_k_*: the screening calculation for the *k*-th screening condition, such as the calculation constraints ‘>’ (more than), ‘<’ (less than), and ‘! = ’ (not in).

factor*_k_*: fold value of screening for the *k*-th screening condition.

inclusion*_k_*: the screening results are optionally inclusive or exclusive, i.e., ‘Yes’ or ‘No’.

Then the information for calculation between different Lib can be defined as follows:
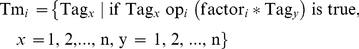
(2)


where Tm is Tag set, Tag*_x_* and Tag*_y_* are cancer-normal pair or normal-cancer pair of the same type of tissue source, Tag*_x_* is the tag expression in one tissue source and Tag*_y_* for another one; *i* is the numbers of different types of tissue sources; and *n* is the total number of attributes of an object.

Then we can define the equation in the form of set theory in the example of 4 different Tm, e.g. Tm*_h_*, Tm*_i_*, Tm*_j_*, and Tm*_k_* for breast, ovary, pancreas, and colon tissues, respectively.
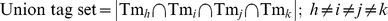
(3)

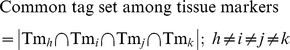
(4)


Tissue-specific tag set among tissue markers 
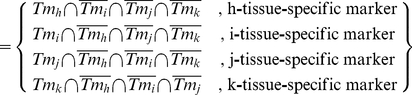
(5)


An example condition is provided in Table S2. Subsequently, the information of the screened matrix data is mined from selected gene maps, clustering (optional), visualization, and output.

### Tag-to-gene module and Tag-to-library module

By inputting the tag sequence, the corresponding gene and SAGE libraries, are screened and matched to the “tag to gene” and “SAGE library” database, respectively.

### Output module

The output module provides filtered matrix, and corresponding information of the matched genes for the SAGE libraries.

## Supporting Information

Figure S1 The 3-layer categorization for SAGE library data. The first layer is 'SAGE technique', the second is 'SAGE library series' and the third is 'SAGE library'.
(0.09 MB DOC)

Table S1 Tag-to-gene database used in this study.
(0.03 MB DOC)

Table S2 Symbolic significance of various filter conditions for A and B*.
(0.03 MB DOC)

Table S3 Features, filter conditions, and symbolic significance of test matrix data.
(0.05 MB DOC)
