# Supplementary material for: hSAGEing: An Improved SAGE-Based Software for Identification of Human Tissue-Specific or Common Tumor Markers and Suppressors
Source: PLoS One. 2010 Dec 17;5(12):e14369. doi: 10.1371/journal.pone.0014369 (PMC3003683; doi:10.1371/journal.pone.0014369)

**Figure S1.** The 3-layer categorization for SAGE library data. The first layer is ‘SAGE technique’, the second is ‘SAGE library series’ and the third is ‘SAGE library’.


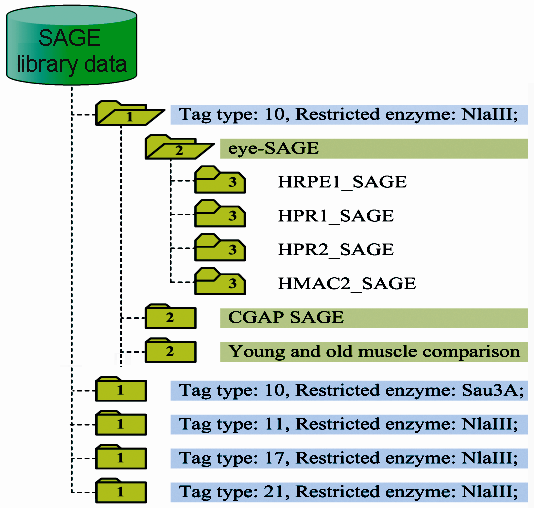

Supplement: Figure S1 — The 3-layer categorization for SAGE library data. The first layer is 'SAGE technique', the second is 'SAGE library series' and the third is 'SAGE library'. (0.09 MB DOC) [file pone.0014369.s001.doc]
