# Supplementary material for: hSAGEing: An Improved SAGE-Based Software for Identification of Human Tissue-Specific or Common Tumor Markers and Suppressors
Source: PLoS One. 2010 Dec 17;5(12):e14369. doi: 10.1371/journal.pone.0014369 (PMC3003683; doi:10.1371/journal.pone.0014369)
Supplement: Table S1 — Tag-to-gene database used in this study. (0.03 MB DOC) [file pone.0014369.s002.doc]

**Table S1. Tag-to-gene database used in this study***

| Database title | Tag type | Restricted enzyme | Last update |
| --- | --- | --- | --- |
| SAGEmap_Hs_NlaIII_10_best | 10 | NlaIII | 2008/10/24 |
| SAGEmap_Hs_Sau3A_10_best | 10 | Sau3A | 2008/10/24 |
| SAGEmap_Hs_NlaIII_17_best | 17 | NlaIII | 2008/10/24 |
| SAGEmap_Hs_Sau3A_17_best | 17 | Sau3A | 2008/10/24 |

***** Mapping data of SAGEmap (<ftp://ftp.ncbi.nlm.nih.gov/pub/sage/mappings/>)
