# Supplementary material for: hSAGEing: An Improved SAGE-Based Software for Identification of Human Tissue-Specific or Common Tumor Markers and Suppressors
Source: PLoS One. 2010 Dec 17;5(12):e14369. doi: 10.1371/journal.pone.0014369 (PMC3003683; doi:10.1371/journal.pone.0014369)
Supplement: Table S2 — Symbolic significance of various filter conditions for A and B*. (0.03 MB DOC) [file pone.0014369.s003.doc]

## Table S2. Symbolic significance of various filter conditions for A and B*

| Condition | Symbolic significance |
| --- | --- |
| A > B (by 2.0) | inclusion=Yes | Find the up-expressing tags for A compared with B. |
| A > B (by 2.0) | inclusion=No | Find the tags which are not up-expressing for A compared with B. |
| A < B (by 2.0) | inclusion=Yes | Find the down-expressing tags for A compared with B. |
| A < B (by 2.0) | inclusion=No | Find the tags which are not down-expressing for A compared with B. |
| A ! ******= B (by 2.0) | inclusion=Yes | Find the undetectable tags for A compared with B. |
| A ! ******= B (by 2.0) | inclusion=No | Find the tags which are not undetectable for A compared with B. |

***** A and B indicate two SAGE libraries for comparison to each other. Usually, these are case and control libraries.

****** A ! indicates the undetectable tags for A; the undetectable tags are whose tags showed the zero count under the total test counts.
