# Supplementary material for: hSAGEing: An Improved SAGE-Based Software for Identification of Human Tissue-Specific or Common Tumor Markers and Suppressors
Source: PLoS One. 2010 Dec 17;5(12):e14369. doi: 10.1371/journal.pone.0014369 (PMC3003683; doi:10.1371/journal.pone.0014369)
Supplement: Table S3 — Features, filter conditions, and symbolic significance of test matrix data. (0.05 MB DOC) [file pone.0014369.s004.doc]

**Table S3. Features, filter conditions, and symbolic significance of test matrix data**

| **(A) SAGE**  **Library** | | **Description** | | **No.**  **of tag** | **Type of tag** |
| --- | --- | --- | --- | --- | --- |
| GSM728 | | SAGE_Colon_normal_B_NC1 (SAGE_NC1); Normal colonic epithelium; Sample type: Bulk [GSM383869]^*1^ | | 50,179 | 17,913 |
| GSM755 | | SAGE_Colon_adenocarcinoma_B_Tu102 (SAGE_Tu102); Colon, primary tumor; Sample type: Bulk [GSM383859] | | 57,686 | 23,001 |
| GSM719 | | SAGE_Ovary_normal_CS_HOSE_4 (SAGE_HOSE_4); Ovary, normal surface epithelium; Sample type: Cell line [GSM383930] | | 48,552 | 16,317 |
| GSM735 | | SAGE_Ovary_adenocarcinoma_B_OVT-6 (SAGE_OVT-6); Ovary, primary ovarian tumour, serous adenocarcinoma.; Sample type: Bulk [GSM383922] | | 42,445 | 18,780 |
| GSM736 | | SAGE_Ovary_adenocarcinoma_B_OVT-7 (SAGE_OVT-7); Ovary, primary tumor. Serous adenocarcinoma.; Sample type: Bulk [GSM383923] | | 55,002 | 19,711 |
| GSM737 | | SAGE_Ovary_adenocarcinoma_B_OVT-8 (SAGE_OVT-8); Ovary, primary ovarian tumour. Serous adenocarcinoma.; Sample type: Bulk [GSM383924] | | 33,675 | 16,661 |
| GSM14770 | | SAGE_Pancreas_normal_B_1(SAGE_Pancreas_normal_B_1); pancreas; normal, Sample type: bulk [GSM383937] | | 21,725 | 8,769 |
| GSM743 | | SAGE_Pancreas_adenocarcinoma_B_91-16113 (SAGE_Panc_91-16113); pancreas, epithelium, ductal, adenocarcinoma; Sample type: bulk [GSM383931] | | 33,957 | 16,004 |
| GSM744 | | SAGE_Pancreas_adenocarcinoma_B_96-6252 (SAGE_Panc_96-6252); pancreas, epithelium, ductal, adenocarcinoma; Sample type: bulk [GSM383932] | | 35,750 | 15,685 |
| GSM677 | | SAGE_Breast_normal_epithelium_BEREP4+_AP_N2 (SAGE_Br_N); Breast, normal luminar mammary epithelium, BerEp4 purified cells; Sample type: Bulk [GSM383831] | | 37,642 | 16,297 |
| GSM670 | | SAGE_Breast_carcinoma_B_95-259 (SAGE_95-259); Breast, grade 3, invasive ductal carcinoma - primary ER-, PR- tumor; Sample type: Bulk [GSM383789] | | 40,223 | 15,436 |
| GSM671 | | SAGE_Breast_metastatic_carcinoma_B_LN1 (SAGE_95-260); Breast, lymph node metastasis. ER-, PR- tumor; Sample type: Bulk [GSM383827] | | 45,673 | 16,074 |
| GSM672 | | SAGE_Breast_carcinoma_B_95-347 (SAGE_95-347); Breast, grade 3, invasive ductal carcinoma - primary ER+ PR+ tumor; Sample type: Bulk [GSM383790] | | 67,386 | 22,727 |
| **(B) Filter condition*^2^** | | | **Symbolic significance** | | |
| 1 | GSM755 > GSM728 (by 2.0) | inclusion=Yes | | Colon-specific markers in adenocarcinoma. | | |
| 2 | GSM735 > GSM719 (by 2.0) | inclusion=Yes | | Ovary-specific markers in adenocarcinoma. | | |
| 3 | GSM736 > GSM719 (by 2.0) | inclusion=Yes | | Ovary-specific markers in adenocarcinoma. | | |
| 4 | GSM737 > GSM719 (by 2.0) | inclusion=Yes | | Ovary-specific markers in adenocarcinoma. | | |
| 5 | GSM743 > GSM14770 (by 2.0) | inclusion=Yes | | Pancreas-specific markers in neoplasia. | | |
| 6 | GSM744 > GSM14770 (by 2.0) | inclusion=Yes | | Pancreas-specific markers in neoplasia. | | |
| 7 | GSM670 > GSM677 (by 2.0) | inclusion=Yes | | Breast-specific markers in carcinoma. | | |
| 8 | GSM671 > GSM677 (by 2.0) | inclusion=Yes | | Breast-specific markers in carcinoma. | | |
| 9 | GSM672 > GSM677 (by 2.0) | inclusion=Yes | | Breast-specific markers in carcinoma. | | |

***^1^** New library no. because GEO renames the CGAP SAGE library no. ***^2^** Condition for inclusion = Yes or No (selectable). All conditions 1 to 9 are considered simultaneously. GSM-series libraries at the left side of “>” belong to the tumor tissue SAGE libraries. The other side of “>” belongs to the control tissue SAGE libraries. Once “No” is selected for inclusion, this condition is not included in the summary of the filter conditions.
